# Multi-objective optimization of light guide column injection molding process using RSM and NSGA‑II with numerical verification

**DOI:** 10.1371/journal.pone.0361371

**Published:** 2026-10-30

**Authors:** Huajun Xu

**Affiliations:** Anhui Technical College of Water Resources and Hydroelectric Power, Hefei, China; Henan Polytechnic University, CHINA

## Abstract

Injection molding technology involves fully melting plastic material through screw agitation at a specific temperature, injecting it into the mold cavity under high pressure, and obtaining the molded part after cooling and solidification. Injection molding offers high production efficiency while meeting the molding demands of complex plastic components. However, enhancing the dimensional accuracy of injection-molded parts has become a critical aspect of this process. The injection-molded components of the XG-99Q2 light guide column were selected as the subject of this study. Initially, three runner system designs were created: “Wang” shape, ‘Gong’ shape, and “Yi” shape configurations. Each design underwent process analysis for its respective injection molding process. Subsequently, six fundamental injection parameters were identified, and an L32 orthogonal experimental design was employed to systematically investigate their effects on two critical objectives: plastic volume shrinkage and warpage deformation. The experimental design incorporated signal-to-noise ratio processing, with weights for total warpage and volume shrinkage derived using the entropy weight method. This approach facilitated qualitative analysis of gray correlation coefficients and correlations among identified factors, revealing significant relationships between injection time, holding pressure, and melt temperature. Prediction models for warpage and shrinkage were developed using the Box-Behnken experimental design from response surface methodology, providing a foundation for comprehensive quantitative analysis. Additionally, the NSGA-II algorithm was applied to multi-objective optimization, yielding a set of Pareto optimal solutions. Finally, the reliability of the method was verified through simulation based validation.

## 1. Introduction

The injection moulding process includes several stages: mould closing, filling, pressure holding, cooling, mould opening, and demolding. Firstly, the plastic raw material needs to be heated to the melting temperature, transforming into a viscous flow state to ensure sufficient flow and filling capabilities. Subsequently, the melt is injected into the mold cavity under the push of the screw, completing the filling and preliminary molding. Maintaining a specific pressure on the molten material in the mould cavity for an appropriate period of time can support the shrinkage process and minimise defects, ultimately helping preserve the quality of the final product. When the plastic melt fills the mould, it initiates the cooling and solidification process. The temperature of the moulded parts will gradually decrease until the specified demolding temperature is reached, enabling safe disassembly [1–4].

During the mold testing phase of injection molding, it is necessary to repeatedly adjust process parameters based on product structure and material characteristics to ensure molding quality. Therefore, reducing the number of mold tests is crucial [5–7]. The core task of process parameter optimization is to determine the parameter combination that achieves optimal production or performance results through experimentation and systematic analysis [8–11]. In recent years, the comprehensive application of modern CAE technology, statistical methods, and optimization algorithms for injection molding process parameter optimization has become an important research direction [12–19]. For example, Kitayama et al. [20] used a radial basis function network combined with a sequential approximate optimization method to determine the Pareto boundaries of fusion line, clamping force, and cycle time; Hong et al. [21] proposed a multi-objective optimization method based on BP neural network and NSGA-II algorithm; Li et al. [22] analyzed the influence of process parameters on the shrinkage rate of PFA parts using Moldflow and orthogonal experiments, finding that injection rate had the most significant impact on tube length shrinkage, while melt temperature, packing pressure, and screw speed mainly affected tube outer diameter shrinkage; Ozcelik and Erzurumlu [23] optimized process parameters to reduce warpage through variance analysis, artificial neural networks, and genetic algorithms; Guo et al. [24] proposed a DNN-GA-MCS strategy to enhance the structural performance of carbon fiber reinforced PA6 injection molded products; Zhai et al. [25] used six optimization algorithms and inverse deformation design to reduce volume shrinkage and warpage; Zhou et al. [26] proposed a differential sensitivity fusion method to systematically optimize process parameters to improve product quality, production efficiency, and control costs.

In addition, Wu et al. [27] combined multi-objective optimization with Moldflow simulation, proposing an effective method for controlling the nonlinear shrinkage of low modulus plastic gears in micro-injection molding. Lee et al. [28] constructed an artificial neural network model based on six key process conditions, achieving accurate prediction of injection molded product performance. This method not only improves prediction accuracy and production efficiency but also significantly reduces the time spent on process debugging. Zeng et al. [29] proposed a multi-objective optimization strategy based on stratified sampling and comprehensive entropy weight to determine the combination of process parameters that minimizes the comprehensive defect value.

Warpage is a common defect in injection molding, which can easily lead to dimensional deviations and assembly difficulties. With the advancement of mold flow analysis technology, scholars both domestically and internationally have conducted extensive research on mold structure improvement and process parameter optimization to balance product quality and cost control, aiming to suppress warpage deformation in thin-walled products [14,30]. This deformation mainly stems from residual stress caused by uneven cooling, excessive injection pressure, and improper melt temperature. To address this, comprehensive control can be achieved by combining the following dimensions: in the product design stage, optimizing the structure to enhance rigidity, maintain uniform wall thickness, and set a reasonable demolding slope; in mold design, arranging cooling water channels reasonably to ensure uniform temperature distribution in the mold cavity [31,32]; in terms of materials and processes, selecting appropriate plastics and increasing the cooling rate can help reduce deformation. In addition, many process optimization studies have been conducted focusing on defects such as warpage, sink marks, and uneven shrinkage [33,34].

This article takes the injection-moulded part of the XG-99Q2 light guide column as the research object and compares three pouring system schemes: the “Wang”-shaped, “Gong”-shaped, and “Yi”-shaped. Six fundamental injection parameters were identified, and an L32 orthogonal experimental design was employed to systematically investigate their effects on two critical objectives: plastic volume shrinkage and warpage deformation. The experimental design incorporated signal-to-noise ratio processing, with weights for total warpage and volume shrinkage derived using the entropy weight method. This approach facilitated qualitative analysis of gray correlation coefficients and correlations among identified factors, revealing significant relationships between injection time, holding pressure, and melt temperature. Prediction models for warpage and shrinkage were developed using the Box-Behnken experimental design from response surface methodology, providing a foundation for comprehensive quantitative analysis. Additionally, the NSGA-II algorithm was applied to multi-objective optimization, yielding a set of Pareto optimal solutions. And use the NSGA-II algorithm for multi-objective optimisation to obtain the Pareto optimal solution set.

## 2. Materials and methods

The plastic part is an XG-99Q2 light guide column, as shown in Fig 1. Its external structural dimensions are 95 mm × 15 mm × 24.9 mm, including multiple independent light guide paths, which require the use of a multi-cavity mould. During injection moulding, it is necessary to control the consistency of each channel to avoid uneven light output due to differences in optical path lengths. Considering the application scenarios of plastic parts, high-transparency materials may require high-precision injection moulds to ensure surface smoothness and structural accuracy; Materials with good temperature resistance may allow higher processing temperatures, thereby optimising moulding parameters; Materials with strong impact resistance may require specialised demolding processes to avoid cracking caused by stress concentration. Therefore, polypropylene material was selected this time. In Moldflow software, a double-layer mesh is selected, which is suitable for plastic parts with thin-walled shells and relatively uniform wall thickness. The total number of mesh elements in the component is 10866, and the total number of mesh elements in the mold is 896235. Fig 2 shows the three-dimensional model and mesh division of the plastic part.

**Fig 1:**
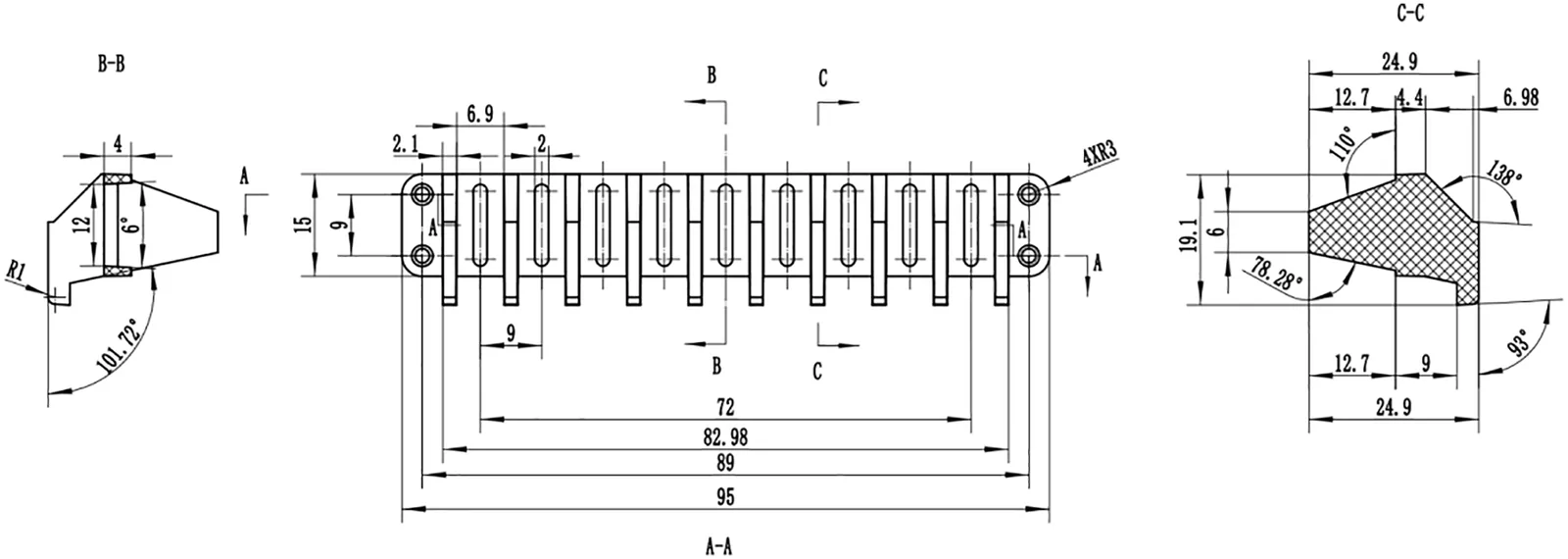
3D model of plastic parts.

**Fig 2:**
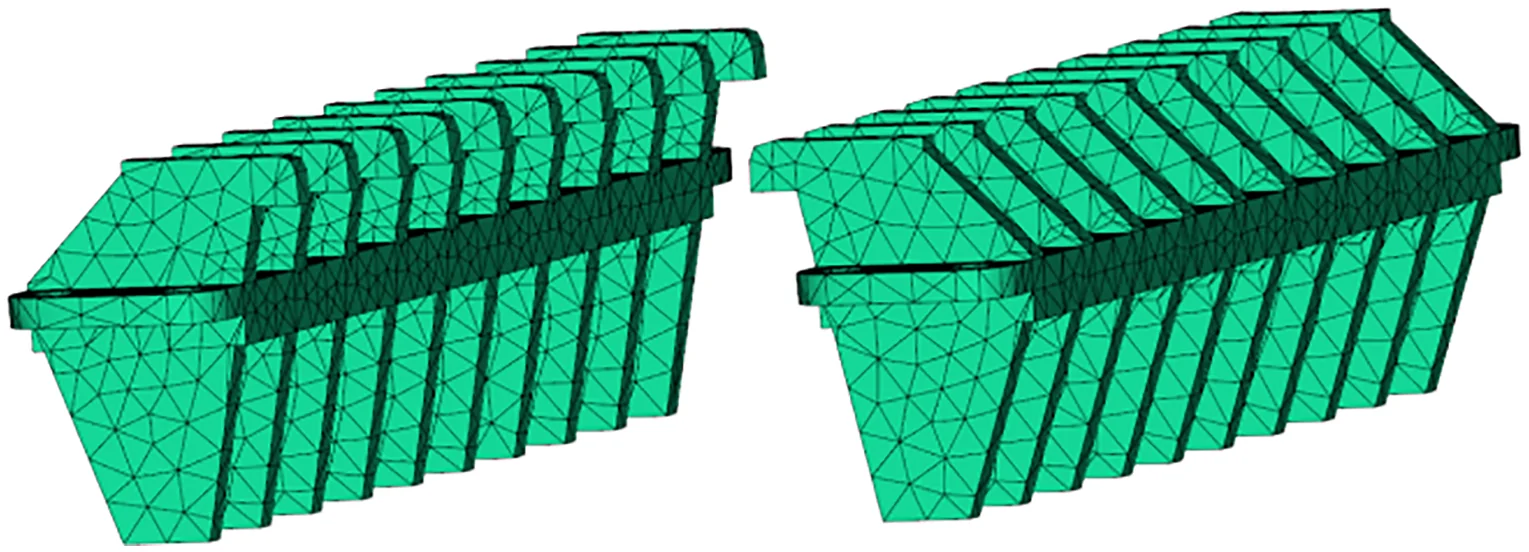
3D modeling and mesh division of plastic parts.

The design of the pouring system has a significant impact on the molding cycle and product quality. Due to its small size and thin wall, a dual cavity mold design is adopted as it can ensure smooth flow of the melt and improve the quality of injection molded parts. The pouring system consists of a cold main runner, a cold runner, and a cold gate. Three types of pouring system designs have been created: “Wang,” “Gong,” and “Yi” configurations. As shown in Fig 3. During the molding process, cooling water or other media circulates through the cooling channels in the mold to ensure uniform cooling, thereby reducing product shrinkage and deformation. Adopting a circular cooling circuit. The overall cooling system model is shown in the Fig 4.

**Fig 3:**
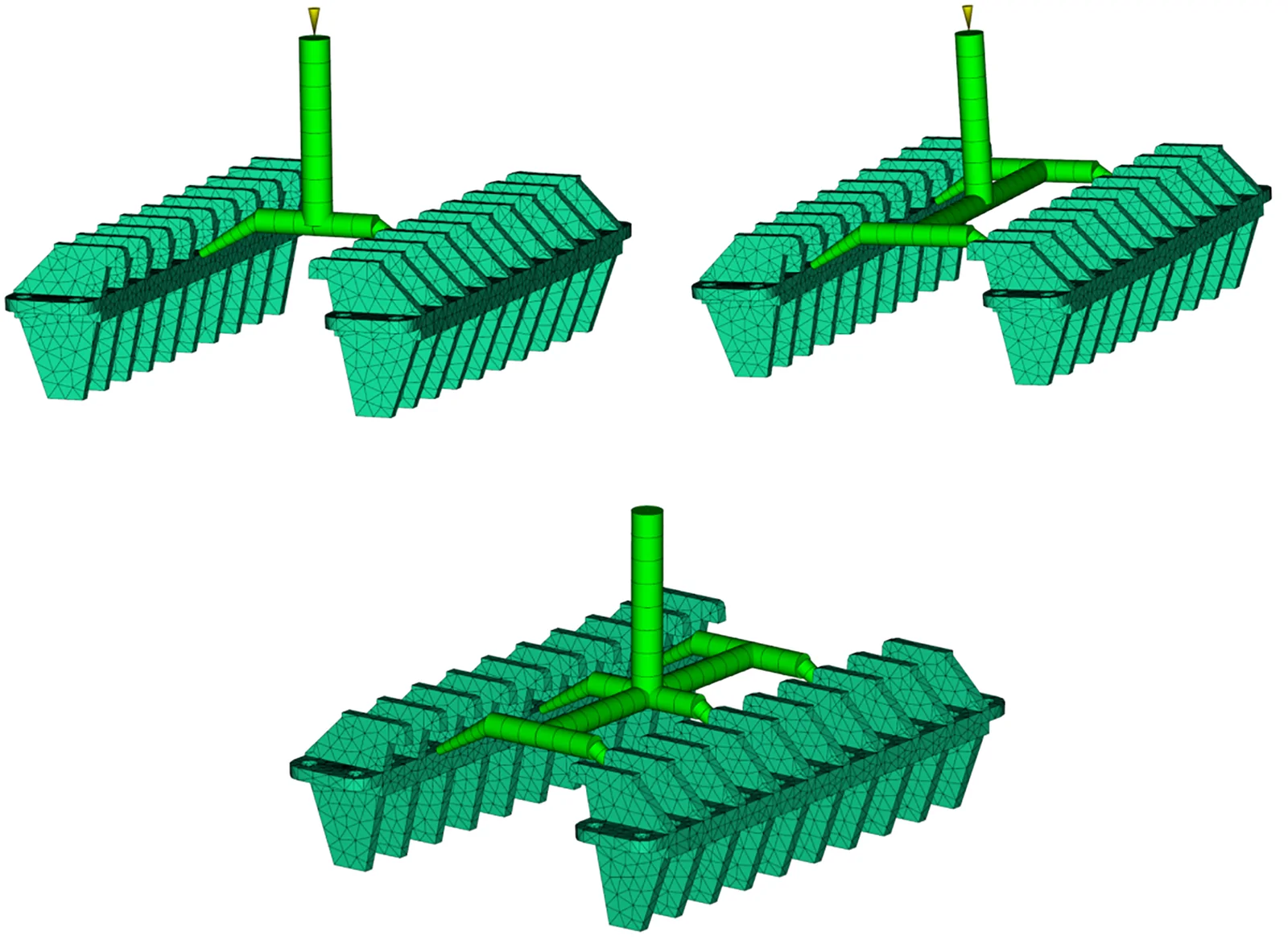
Three types of pouring systems (a) “Wang” type (b) “Gong” type (c) “Yi” type.

**Fig 4:**
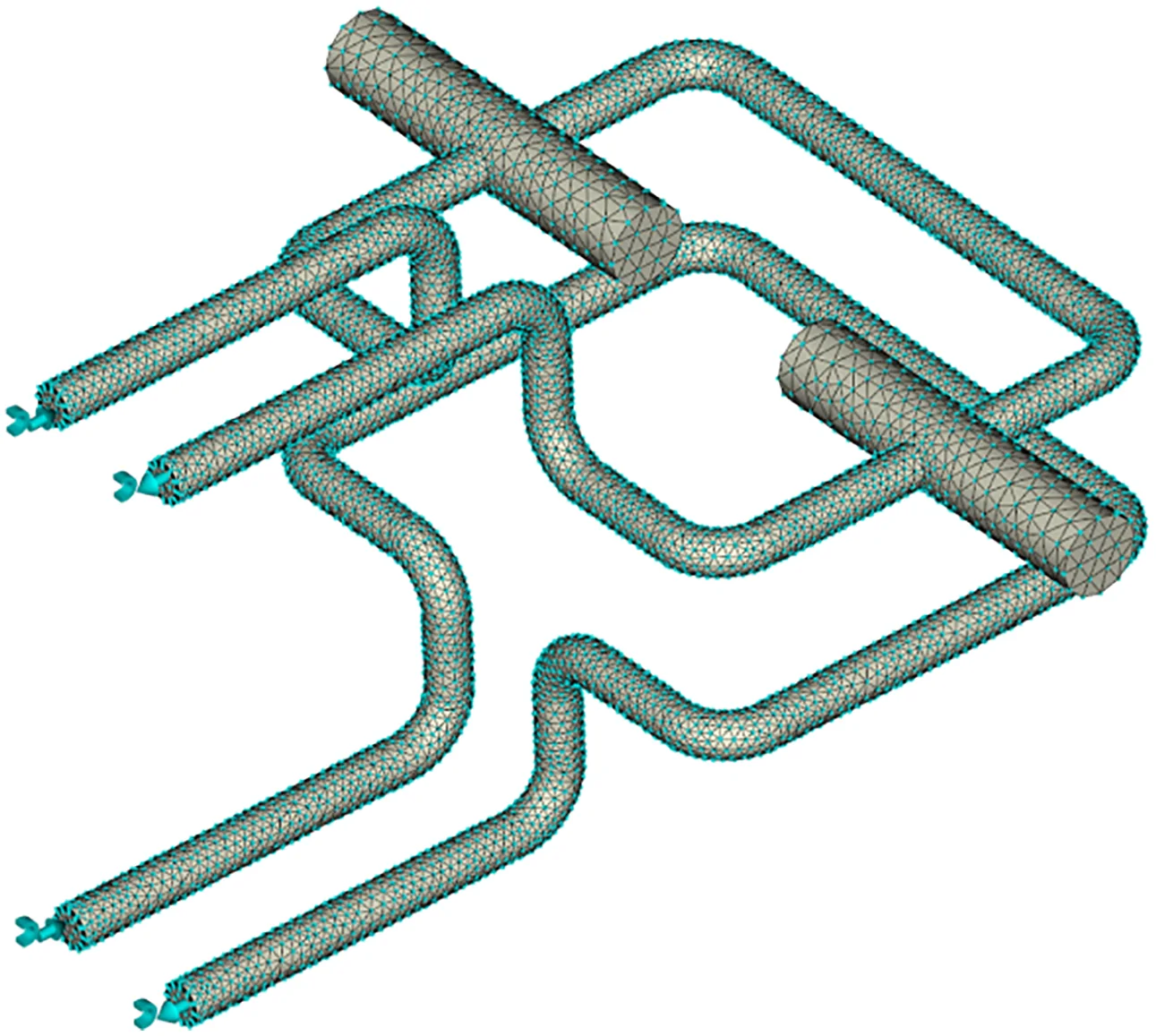
Overall cooling system model.

### 2.1. Orthogonal experimental design

The process parameters involved in this study (melt temperature, mold opening time, injection time, packing time, packing pressure, and mold temperature) are detailed in Table 1; the corresponding experimental results for volumetric shrinkage and total warpage deformation are summarized in Table 2.

**Table 1:** Orthogonal test factor level table.

Factor	Level			
	1	2	3	4
A. melt temperature (℃)	210	220	230	240
B. mould opening time (s)	3	4	5	6
C. injection time (s)	1.0	1.5	2.0	2.5
D. holding time (s)	8	10	12	14
E. holding pressure (MPa)	70	80	90	100
F. mould temperature (℃)	50	60	70	80

**Table 2:** Orthogonal experiment scheme and results.

No.	Process parameters						Results	
	melt temperature (℃)	mould opening time (s)	injection time (s)	holding time (s)	holding pressure (MPa)	mould temperature (℃)	Warpage deformation /mm	Shrinkage rate /%
1	210	3	1	8	70	50	0.1288	16.15
2	210	4	1.5	10	80	60	0.1254	15.75
3	210	5	2	12	90	70	0.1653	15.41
4	210	6	2.5	14	100	80	0.1912	15.15
5	220	3	1	10	80	70	0.1316	16.67
6	220	4	1.5	8	70	80	0.1316	16.45
7	220	5	2	14	100	50	0.1943	16.09
8	220	6	2.5	12	90	60	0.1715	15.78
9	230	3	1.5	12	100	50	0.1884	16.94
10	230	4	1	14	90	60	0.1639	10.74
11	230	5	2.5	8	80	70	0.1473	16.49
12	230	6	2	10	70	80	0.1370	16.81
13	240	3	1.5	14	90	70	0.1646	17.52
14	240	4	1	12	100	80	0.1855	10.39
15	240	5	2.5	10	70	50	0.1374	16.83
16	240	6	2	8	80	60	0.1422	17.31
17	210	3	2.5	8	100	60	0.1927	15.04
18	210	4	2	10	90	50	0.1652	15.51
19	210	5	1.5	12	80	80	0.1250	15.80
20	210	6	1	14	70	70	0.1286	12.17
21	220	3	2.5	10	90	80	0.1716	15.82
22	220	4	2	8	100	70	0.1944	16.10
23	220	5	1.5	14	70	60	0.1286	12.17
24	220	6	1	12	80	50	0.1319	11.08
25	230	3	2	12	70	60	0.1368	16.83
26	230	4	2.5	14	80	50	0.1473	16.51
27	230	5	1	8	90	80	0.1642	17.23
28	230	6	1.5	10	100	70	0.1883	16.86
29	240	3	2	14	80	80	0.1428	17.26
30	240	4	2.5	12	70	70	0.1375	16.74
31	240	5	1	10	100	60	0.1859	17.72
32	240	6	1.5	8	90	50	0.1650	17.51

### 2.2. Signal-to-noise ratio and normalization processing

The signal-to-noise ratio expression is shown in Equation (1).


$$\begin{array}{c} \frac{S}{N}=-10lg(\frac{\text{1}}{n}\sum_{i=1}^{n}x_{i}{)}^{\text{2}} \end{array}$$ (1)

where *S/N* represents the signal-to-noise ratio, *n* is the number of repeated experiments, and *x*_*i*_ is the result of the ith experiment.

To ensure the equivalence and orderliness of the data, normalization processing must be carried out as shown in Equation (2).


$$\begin{array}{c} y_{i}=\frac{max\left(\eta_{i}\right)-\eta_{i}}{max\left(\eta_{i}\right)-min\left(\eta_{i}\right)} \end{array}$$ (2)

where, $\eta_{i}$ is the signal-to-noise ratio after the i-th experiment data conversion; $y_{i}$ is the normalized value of $\eta_{i}$.

### 2.3. Grey correlation coefficient

Based on the processed data series, the grey correlation coefficient ($\zeta_{i}$) can be calculated as shown in Equation (3).


$$\begin{array}{c} \zeta_{i}=\frac{min\left|y_{i}^{o}-y_{i}\right|+\rho max\left|y_{i}^{o}-y_{i}\right|}{\left|y_{i}^{o}-y_{i}\right|+\rho max\left|y_{i}^{o}-y_{i}\right|} \end{array}$$ (3)

where, $y_{i}^{o}$ is the normalized ideal value of the data corresponding to the i-th experiment; ρ is the resolution coefficient, with a value of 0.5.

### 2.4. Calculate the weight of multiple indicators

The total warpage deformation of plastic parts directly affects the molding dimensional tolerance, thereby determining the assembly fit accuracy of the product; while the volume shrinkage rate is a key factor affecting surface quality. To scientifically evaluate the above indicators, this paper introduces a method combining entropy weight analysis and grey relational analysis, determining the weight coefficient of each indicator based on its entropy value. The larger the entropy value, the smaller the variability of the indicator, and the corresponding weight in the evaluation decreases accordingly. The implementation steps of this method can be summarized as follows:

(1) Using the data in Table 2 as input, apply formula (4) to calculate and construct a decision matrix for evaluating the comprehensive quality results.

$$\begin{array}{c} X_{m\times n}=\left[\begin{array}{cccc} @cccc@X_{11} & X_{12} & \cdots & X_{1n}\\ X_{21} & X_{22} & \cdots & X_{2n}\\ \vdots & \vdots & \vdots & \vdots \\ X_{m1} & X_{m2} & \cdots & X_{mn} \end{array}\right] \end{array}$$ (4)

where, m is the number of experiments (m = 32), n is the experimental index (n = 2).

(2) Further regularize the decision matrix as shown in Equation (5).

$$\begin{array}{c} \lambda_{ij}=\frac{X_{ij}}{\sqrt{\sum_{i=1}^{m}X_{ij}^{\text{2}}}} \end{array}$$ (5)

where, $\lambda_{ij}$ is the regularization result of the jth test indicator in the i-th experiment.

(3) Calculate the entropy value by normalizing the data as shown in Equation (6).

$$\begin{array}{c} E_{j}=-\frac{\text{1}}{lnm}\sum_{i=1}^{m}\frac{\lambda_{ij}}{\sum_{i=1}^{m}\lambda_{ij}}ln\frac{\lambda_{ij}}{\sum_{i=1}^{m}\lambda_{ij}} \end{array}$$ (6)

where, J is the experimental index (j = 2), i is the number of experiments (i = 32), and m is the number of experiments (m = 32).

(4) Calculate the weight coefficients using Equation (7).

$$\begin{array}{c} \omega_{i}=\frac{1-E_{j}}{\sum_{j=1}^{n}1-E_{j}}\times 100\% \end{array}$$ (7)

where, J is the experimental indicator (j = 2), and n is the number of experiments (n = 32).

### 2.5. Grey correlation calculation

After obtaining the weight coefficients through the above calculation, the grey correlation degree is calculated as shown in Equation (8).


$$\begin{array}{c} \gamma_{i}=\sum_{j=1}^{\text{2}}\xi_{ij}\omega_{j} \end{array}$$ (8)

where, $\xi_{ij}$ is the grey correlation coefficient, j is the experimental index (j = 2), and i is the number of experiments (i = 32).

## 3. Results and discussion

### 3.1. Analysis of different types of gates

Fig 5 compares the warpage deformation of injection molded parts under three gate designs: Wang type, Gong type, and Yi type. The results showed that Gong type gate had the smallest warpage (0.5005 mm), followed by Wang type (0.5276 mm), and Yi type had the largest (0.5439 mm). Compared with the other two types of gates, the warpage value of Gong type gate decreased by about 5.1% (relative to Wang type) and 8.0% (relative to Yi type), respectively. Therefore, under the mold and process conditions studied in this article, the Gong type gate has been determined as the optimal gate structure, and subsequent multi-objective optimization should be based on this gate form.

**Fig 5:**
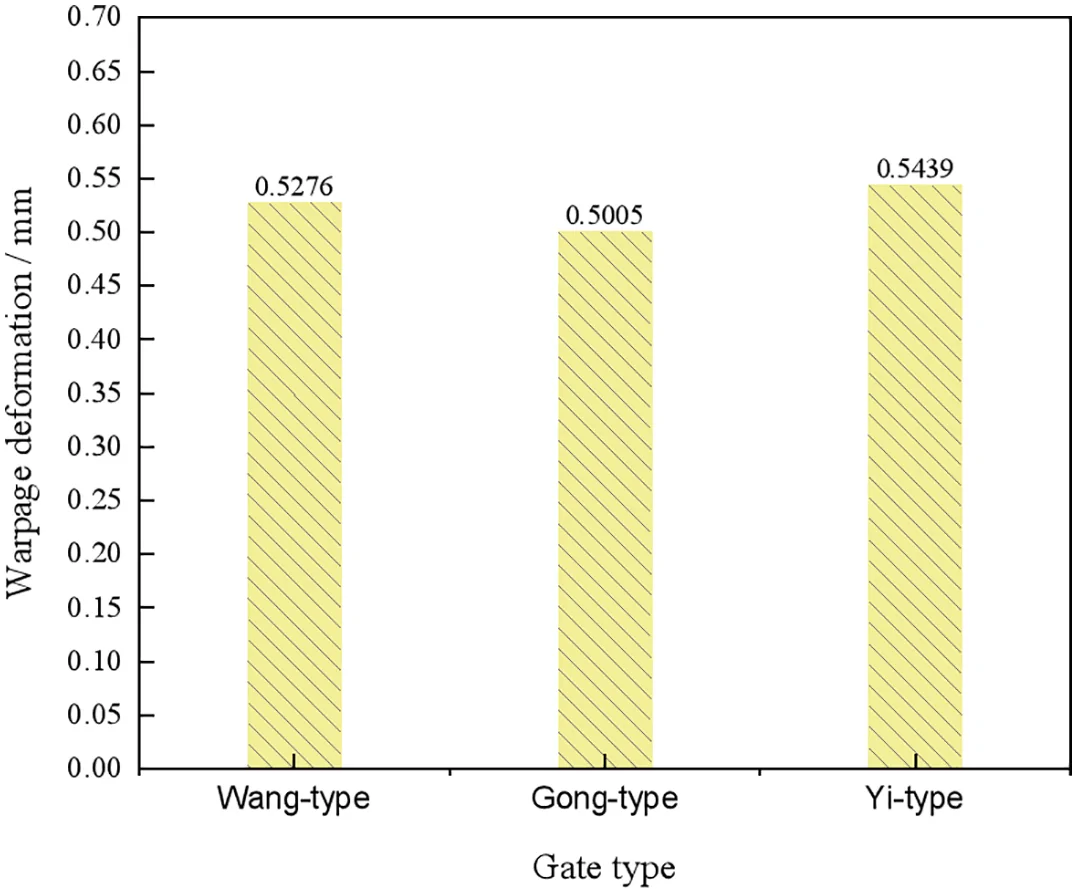
The warpage deformation of different gates.

### 3.2. Analysis of grey correlation

Based on the data in Table 2, the signal-to-noise ratio, non-dimensionalized value, and grey relational degree were calculated using formulas (1) to (3) (Table 3). Further, by substituting the grey relational degree into formulas (4) to (8), the weights of warping deformation and volume shrinkage rate were ultimately determined to be 53.1% and 46.9%, respectively.

**Table 3:** Calculate data for each parameter.

No.	Total amount of warping deformation					Shrinkage depth					$\gamma_{i}$
	*S/N*	*y*_*i*_	$\delta_{i}$	Entropy	Weight	*S/N*	*y*_*i*_	$\delta_{i}$	Entropy	Weight	
1	17.802	0.068	0.881	0.981	0.531	−24.163	0.826	0.377	0.983	0.469	0.645
2	18.034	0.007	0.986			−23.946	0.779	0.391			0.707
3	15.635	0.633	0.441			−23.756	0.738	0.404			0.424
4	14.370	0.962	0.342			−23.608	0.706	0.414			0.376
5	17.615	0.117	0.811			−24.439	0.886	0.361			0.600
6	17.615	0.117	0.811			−24.323	0.861	0.367			0.603
7	14.231	0.999	0.334			−24.131	0.819	0.379			0.355
8	15.315	0.716	0.411			−23.962	0.783	0.390			0.401
9	14.498	0.929	0.350			−24.578	0.916	0.353			0.351
10	15.708	0.614	0.449			−20.620	0.062	0.890			0.655
11	16.636	0.372	0.574			−24.344	0.865	0.366			0.476
12	17.266	0.208	0.707			−24.511	0.901	0.357			0.543
13	15.671	0.623	0.445			−24.871	0.979	0.338			0.395
14	14.633	0.894	0.359			−20.332	0.000	1.000			0.659
15	17.240	0.214	0.700			−24.522	0.903	0.356			0.539
16	16.942	0.292	0.631			−24.766	0.956	0.343			0.496
17	14.302	0.980	0.338			−23.545	0.693	0.419			0.376
18	15.640	0.631	0.442			−23.812	0.750	0.400			0.422
19	18.062	0.000	1.000			−23.973	0.785	0.389			0.714
20	17.815	0.064	0.886			−21.706	0.296	0.628			0.765
21	15.310	0.718	0.411			−23.984	0.788	0.388			0.400
22	14.226	1.000	0.333			−24.137	0.820	0.379			0.355
23	17.815	0.064	0.886			−21.706	0.296	0.628			0.765
24	17.595	0.122	0.804			−20.891	0.120	0.806			0.805
25	17.278	0.204	0.710			−24.522	0.903	0.356			0.544
26	16.636	0.372	0.574			−24.355	0.868	0.366			0.476
27	15.693	0.618	0.447			−24.726	0.947	0.345			0.400
28	14.503	0.928	0.350			−24.537	0.907	0.355			0.353
29	16.905	0.301	0.624			−24.741	0.951	0.345			0.493
30	17.234	0.216	0.698			−24.475	0.893	0.359			0.539
31	14.614	0.899	0.357			−24.969	1.000	0.333			0.346
32	15.650	0.629	0.443			−24.866	0.978	0.338			0.394

Based on the grey relational degree calculated from Table 3, further range analysis was conducted, and the results are summarized in Table 4. The analysis indicates that a larger range R value indicates a more significant impact of the factor level on the quality index.

**Table 4:** Range analysis of grey relational degree.

No.	Factors					
	melt temperature (℃)	mould opening time (s)	injection time (s)	holding time (s)	holding pressure (MPa)	mould temperature (℃)
K1	0.554	0.476	0.609	0.468	0.618	0.498
K2	0.536	0.552	0.486	0.489	0.596	0.536
K3	0.475	0.502	0.454	0.555	0.436	0.488
K4	0.483	0.517	0.448	0.535	0.396	0.523
R	0.079	0.077	0.161	0.087	0.222	0.048

According to the range analysis results in Table 4, the impact of the six factors on the grey relational degree is ranked from small to large as follows: F < B < A < D < C < E. The corresponding optimized combination of molding process parameters is A3B1C4D1E4F3, as shown in Fig 6. The figure illustrates that holding pressure (factor E) is a crucial parameter in the injection molding process. Within a certain process range, increasing the holding pressure helps improve the quality of the molded parts. Moderately increasing the holding pressure can reduce the flow resistance and filling fluctuation of the melt in the mold cavity, thereby mitigating deformation and enhancing molding stability. In addition, the injection time and mold temperature also have a significant impact on warpage deformation; in contrast, the influence of mold opening time and mold temperature are relatively weaker.

**Fig 6:**
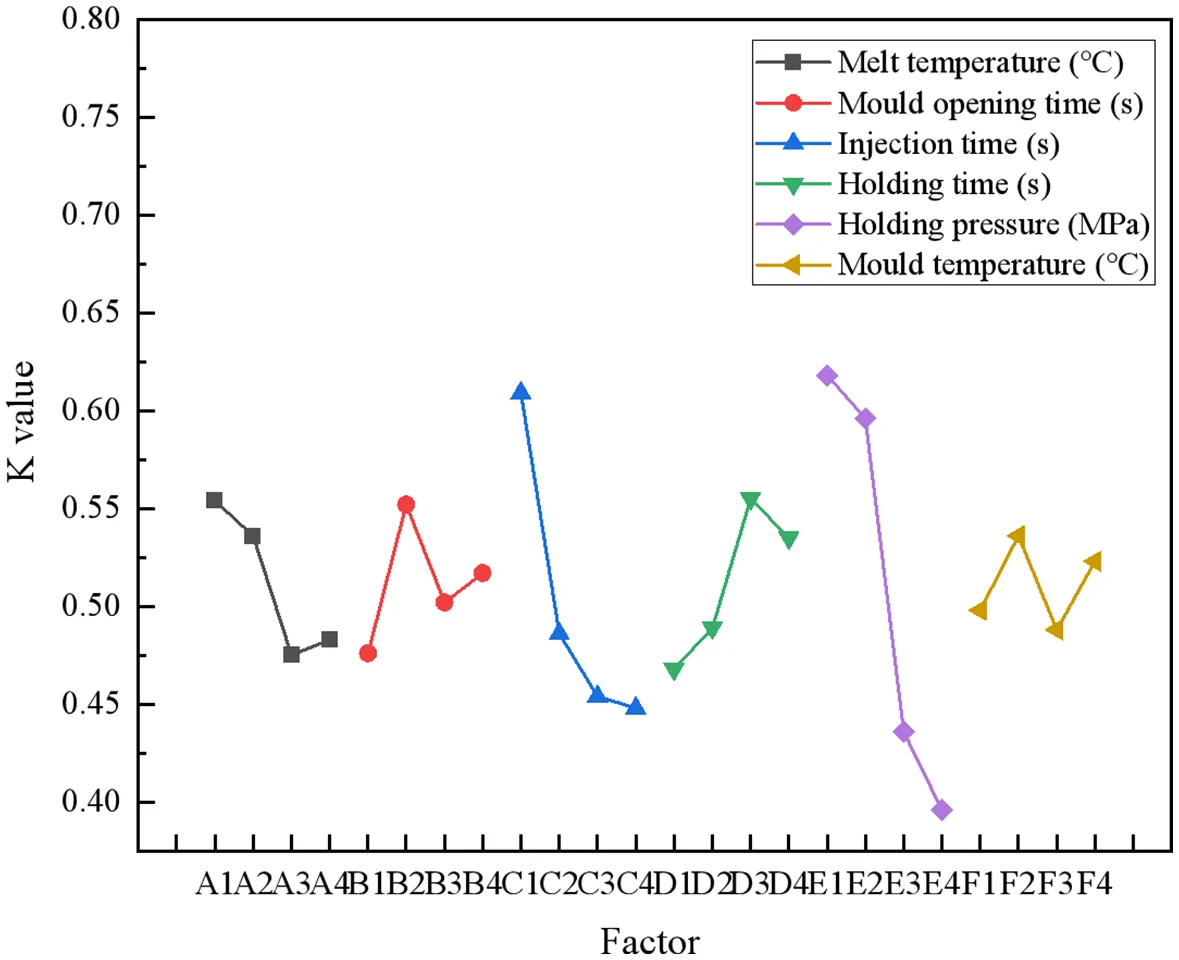
The influence of factor level and K value.

Through comprehensive analysis, it is found that the key to reducing the total amount of warping deformation and volume shrinkage rate lies in regulating the main factors with significant differences such as melt temperature and holding pressure, while synergistically optimizing injection time and mold temperature. The mold opening time and holding pressure time can be flexibly adjusted as auxiliary parameters. By balancing the levels of various factors, the molding quality and production stability of injection molded products can be significantly improved.

### 3.3. Analysis of response surface methodology results

As shown in Table 5, the distance between the cooling channel and the plastic part, the temperature of the cooling medium, and the cooling flow rate were selected as factors, and the warpage deformation was taken as the optimization objective. The model was simulated using the response surface test design method, and the experimental data are shown in Table 6.

**Table 5:** Factor level table.

Level	Factors		
	injection time (s)	holding time (s)	holding pressure (MPa)
−1	2.0	10	90
0	2.5	12	100
1	3.0	14	110

**Table 6:** Experiment design table.

No.	Process parameters			Results	
	injection time (s)	holding time (s)	holding pressure (MPa)	$\gamma_{\alpha }$	$\gamma_{\beta }$
1	2	10	100	16.66	0.1964
2	3	10	100	16.11	0.2027
3	2	14	100	16.66	0.1958
4	3	14	100	16.11	0.2027
5	2	12	90	16.71	0.1693
6	3	12	90	16.18	0.179
7	2	12	110	16.57	0.2266
8	3	12	110	16.05	0.2289
9	2.5	10	90	16.45	0.178
10	2.5	14	90	16.45	0.1778
11	2.5	10	110	16.35	0.2319
12	2.5	14	110	16.35	0.2319
13	2.5	12	100	16.4	0.2036
14	2.5	12	100	16.37	0.2037
15	2.5	12	100	16.4	0.2036
16	2.5	12	100	16.32	0.2033
17	2.5	12	100	16.39	0.2036

Based on the experimental data obtained using response surface analysis, Design Expert software was employed for modeling and analysis to investigate the effects of the distance between the cooling channel and the plastic part, the temperature of the cooling medium, and the cooling flow rate on warpage deformation. The established regression equation models are presented in Equations (9) and (10). The variance analysis results for volumetric shrinkage rate and warpage deformation are listed in Tables 7 and 8, respectively.

**Table 7:** Volume shrinkage rate regression analysis of variance table.

Source	Sum of Squares	df	Mean Square	F-value	p-value	
Model	0.6070	9	0.0674	86.70	< 0.0001	significant
C	0.5778	1	0.5778	742.83	< 0.0001	
D	0.0000	1	0.0000	0.0000	1.0000	
E	0.0276	1	0.0276	35.50	0.0006	
CD	0.0000	1	0.0000	0.0000	1.0000	
CE	0.0000	1	0.0000	0.0321	0.8628	
DE	0.0000	1	0.0000	0.0000	1.0000	
C²	0.0002	1	0.0002	0.2466	0.6347	
D²	0.0010	1	0.0010	1.34	0.2845	
E²	0.0003	1	0.0003	0.3684	0.5630	
Residual	0.0054	7	0.0008			
Lack of Fit	0.0009	3	0.0003	0.2729	0.8428	not significant
Pure Error	0.0045	4	0.0011			
Cor Total	0.6124	16				

**Table 8:** Warping deformation regression analysis of variance table.

Source	Sum of Squares	df	Mean Square	F-value	p-value	
Model	0.0060	9	0.0007	12457.06	< 0.0001	significant
C	0.0001	1	0.0001	1493.71	< 0.0001	
D	8.000E-08	1	8.000E-08	1.51	0.2595	
E	0.0058	1	0.0058	1.089E + 05	< 0.0001	
CD	9.000E-08	1	9.000E-08	1.69	0.2343	
CE	0.0000	1	0.0000	257.61	< 0.0001	
DE	1.000E-08	1	1.000E-08	0.1882	0.6775	
C²	0.0001	1	0.0001	1302.79	< 0.0001	
D²	4.642E-08	1	4.642E-08	0.8735	0.3811	
E²	8.792E-06	1	8.792E-06	165.43	< 0.0001	
Residual	3.720E-07	7	5.314E-08			
Lack of Fit	2.800E-07	3	9.333E-08	4.06	0.1047	not significant
Pure Error	9.200E-08	4	2.300E-08			
Cor Total	0.0060	16				


$$\begin{array}{c} Y_{\alpha }=+19.65550-0.452500C-0.094500D-0.023625E-1.90132E-15CD+0.000500CE \end{array}$$

$$\begin{array}{c} -2.06840E-18DE-0.027000C^{\text{2}}+0.003937D^{\text{2}}+0.000083E^{\text{2}} \end{array}$$ (9)

$$\begin{array}{c} Y_{\beta }=-0.126245+0.122600C-0.000045D+0.000695E+0.0001505CD-0.000370CE \end{array}$$

$$\begin{array}{c} +2.50000\text{E}-06DE-0.016220C^{\text{2}}-0.000026D^{\text{2}}+0.000014E^{\text{2}} \end{array}$$ (10)

The predicted and actual values of volume shrinkage rate and warpage deformation are shown in Fig 7 (a) and 7(b), respectively. From the figure, it can be seen that the predicted values of the response surface model are basically consistent with the experimental values, and the scattered points are roughly distributed along the diagonal, indicating that the model has a good fitting effect.

**Fig 7:**
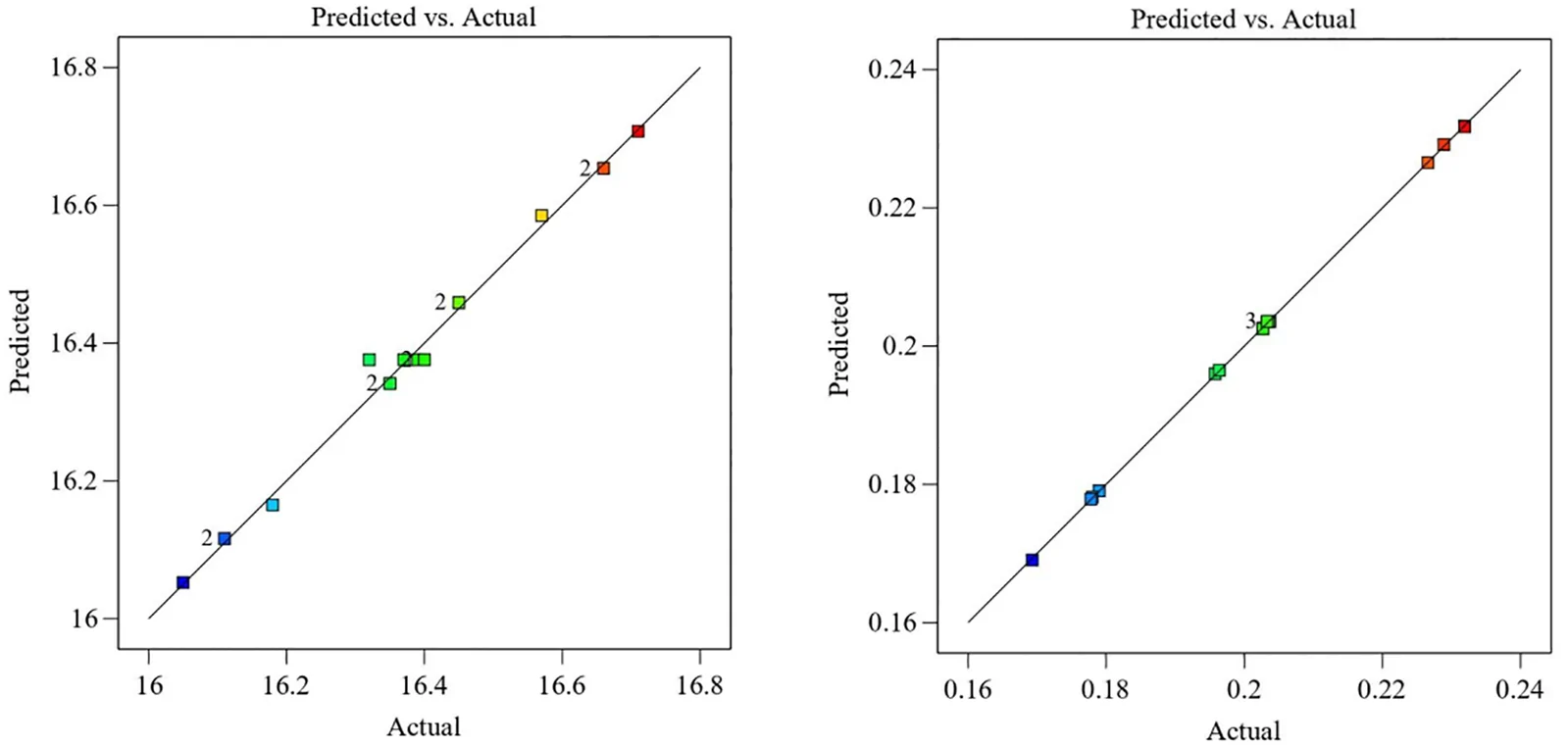
Predicted value and actual value (a) Volume shrinkage rate (b) Warping deformation.

### 3.4. Analysis of NSGA-II optimization results

The problem can be described using Equation (11). Based on the network model established in the previous chapter, the multi-objective optimization problem of volume shrinkage and warping deformation involved in this article will be addressed. The injection molding process parameters will be optimized using the Non dominated Sorting Genetic Algorithms algorithm, and the optimal solution will be obtained.


$$\begin{array}{c} \left\{ \begin{array}{c} \text{Min}Y_{\text{1}}\;(X_{\text{1}},\;X_{\text{2}},\;X_{\text{3}})\\ \text{Min}Y_{\text{2}}\;(X_{\text{1}},\;X_{\text{2}},\;X_{\text{3}}) \end{array}\right. \end{array}$$

$$\begin{array}{c} \left\{ \begin{array}{c} @c\text{2}\text{0}\text{0}\;\circ \text{C}\leq X_{\text{1}}\leq \text{2}\text{3}\text{0}\circ \text{C}\\ \text{2}.\text{0}\text{ s}\leq X_{\text{2}}\leq \text{3}.\text{0}\text{ s}\\ \text{9}\text{0}\text{ MPa }\leq X_{\text{3}}\leq \text{1}\text{1}\text{0}\text{ MPa} \end{array}\right. \end{array}$$ (11)

where *Y*_*1*_ / *Y*_*2*_ represent the function objective value (warping deformation and volume shrinkage rate), *X*_*1*_, *X*_*2*_ and *X*_*3*_ are melt temperature, injection time, holding pressure respectively.

Write the relevant parameters of the algorithm on the MATLAB platform, as shown in Table 9, and solve Equation (11) based on the NSGA-II algorithm. The Pareto optimal frontier obtained by running the NSGA-II algorithm program in MATLAB for 400 iterations is shown in Fig 8. To verify the convergence of the Pareto optimal solution, 1000 random sampling points within the design space were conducted to validate the results of the Pareto front, as shown in Fig 9.

**Table 9:** Comparison of NSGA-Ⅱ algorithm optimization and simulation results.

Factors	Value
Number of constraint variables	3
Output quantity	2
Initial population size	50
Number of iterations	400
Cross probability	0.9
Mutation probability	0.1

**Fig 8:**
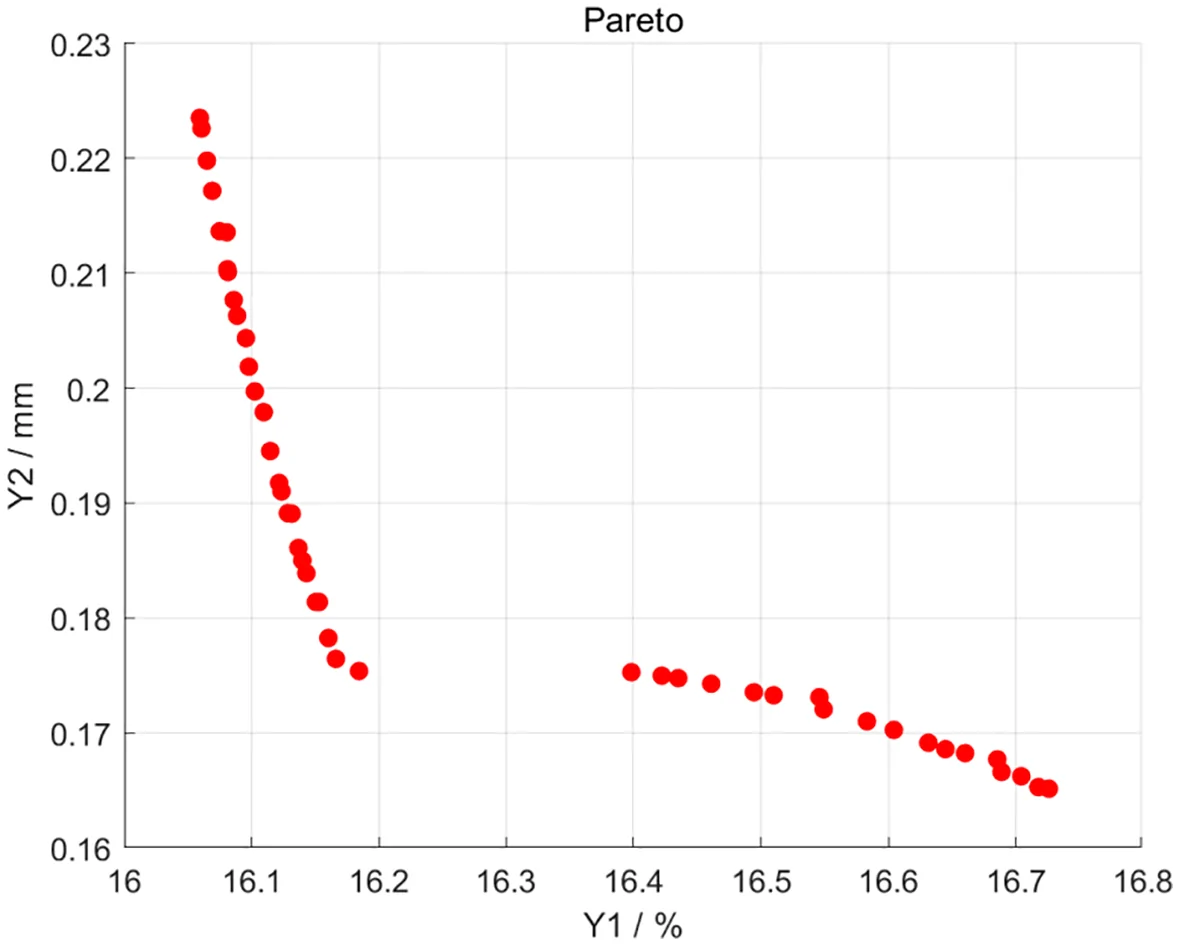
Pareto front.

**Fig 9:**
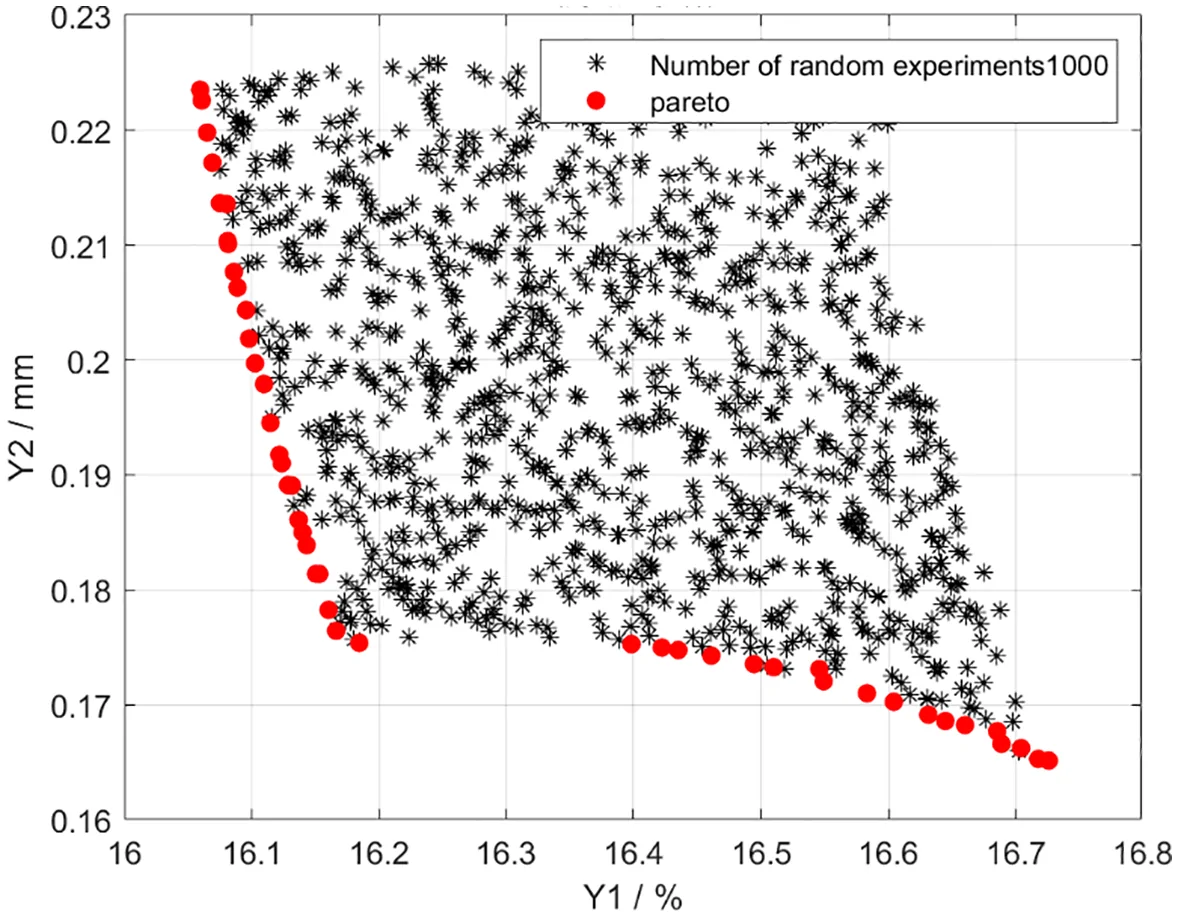
Pareto front validation with 1000 random experiments.

The process parameters optimized based on the NSGA-II algorithm were set in Moldfow, and the simulation results shown in Fig 10 were obtained: the volume shrinkage rate was 9.289%, and the total warpage deformation value was 0.3401 mm.

**Fig 10:**
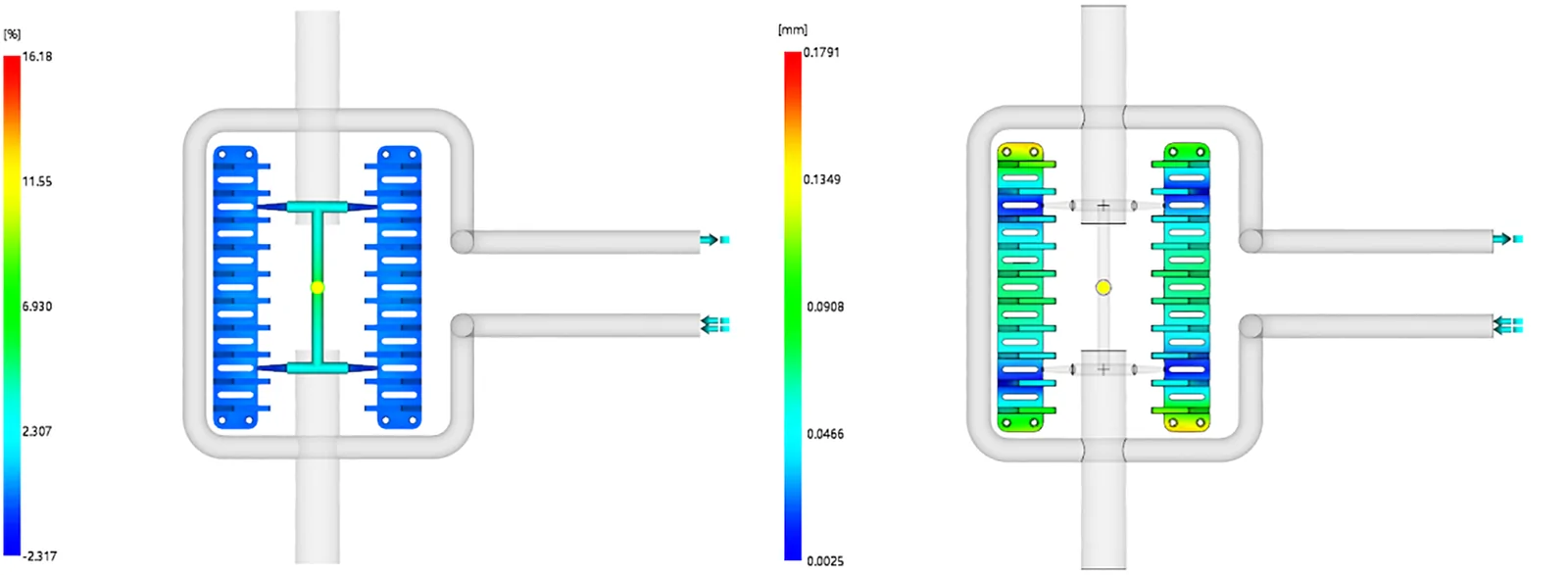
NSGA-Ⅱ optimization parameter simulation results.

### 3.5. Experimental verification

The errors between the algorithm optimization results and the simulation results are shown in Table 10. It can be seen that the various errors between the optimization results and the simulation results are 0.028% and 2.066%, respectively, which proves the reliability of the optimized results.

**Table 10:** Comparison of NSGA-Ⅱ algorithm optimization and simulation results.

	*Y*_*1*_ / %	*Y*_*2*_ / mm
NSGA-II optimization results	16.1845	0.1754
Moldflow simulation results	16.1800	0.1791
Error	0.028	2.066

## 4. Conclusions

Three types of pouring systems were designed for the XG-99Q2 light guide column injection molded parts, including the “Wang”-shaped, “Gong”-shaped, and “Yi”-shaped. The simulation results show that the warpage deformation of the Gong-shaped runner is reduced by 5.1% and 8.0% compared to the “Wang”-shaped, “”Yi -shaped gates, respectively.

Based on this, an orthogonal experiment with four levels and six factors was conducted, and the entropy weight method was used to determine the weights of total warpage and volume shrinkage rate, thereby calculating the grey relational degree. Through grey relational analysis, the influence of various process parameters on quality indicators was systematically evaluated. The analysis showed that the impact of the six factors on the grey relational degree is ranked from small to large as follows: F < B < A < D < C < E. The corresponding optimized combination of molding process parameters is A3B1C4D1E4F3.

The various errors between the optimized results and the simulation results are 0.028% and 2.066%, respectively, which proves the reliability of the optimized results. The combination of qualitative and quantitative analysis methods with NSGA-II algorithm can significantly improve the quality defects of plastic parts in the injection molding process.

The verification results based on simulated numerical support show that combining qualitative analysis with quantitative analysis and NSGA-II algorithm has great potential in reducing the occurrence of quality defects in plastic parts during injection molding process. Although these findings provide a promising way to reduce defects in injection molding, current evidence does not support universal applicability across all part types and production scales. Before recommending this method for industrial practice, it is necessary to conduct replication and long-term stability testing under a wider range of conditions.

## 5. Research limitations

This case study is based on a specific light guide column, so caution should be exercised when generalizing the conclusion to other scenarios. This study mainly verified the warpage deformation and volume shrinkage rate of plastic parts. The number of repetitions of the validation experiment is relatively limited, which fails to cover the variability in long-term operation. The boundary conditions used in injection molding simulation have inherent approximations, which may affect the deviation between optimization results and actual conditions.

The optimization conclusion of this article is currently limited to pure numerical verification. In future research, we plan to conduct trial molds on actual injection molding machines and conduct high-precision quantitative measurements of the warpage, shrinkage rate, and residual stress of the molded parts (such as using a coordinate measuring instrument and X-ray diffraction) to further support the effectiveness of this strategy in physical environments.

## Supporting information

S1 File Data. (XLSX)
